# Big Effects of Small RNAs: A Review of MicroRNAs in Anxiety

**DOI:** 10.1007/s12035-012-8374-6

**Published:** 2012-11-13

**Authors:** Stefanie Malan-Müller, Sîan Megan Joanna Hemmings, Soraya Seedat

**Affiliations:** 1 Department of Psychiatry, Faculty of Medicine and Health Sciences, Stellenbosch University, Francie van Zijl Drive, Tygerberg, 7505 South Africa; 2 MRC Centre for Molecular and Cellular Biology, Division of Molecular Biology and Human Genetics, Faculty of Medicine and Health Sciences, Stellenbosch University, P.O. Box 19063, Francie van Zijl Drive, Tygerberg, 7505 South Africa; 3 Department of Psychiatry, Stellenbosch University, P.O. Box 19063, Tygerberg, 7505 South Africa

**Keywords:** MicroRNAs, Anxiety disorders, Panic disorder, Posttranscriptional regulation

## Abstract

Epigenetic and regulatory elements provide an additional layer of complexity to the heterogeneity of anxiety disorders. MicroRNAs (miRNAs) are a class of small, noncoding RNAs that have recently drawn interest as epigenetic modulators of gene expression in psychiatric disorders. miRNAs elicit their effects by binding to target messenger RNAs (mRNAs) and hindering translation or accelerating degradation. Considering their role in neuronal differentiation and synaptic plasticity, miRNAs have opened up new investigative avenues in the aetiology and treatment of anxiety disorders. In this review, we provide a thorough analysis of miRNAs, their targets and their functions in the central nervous system (CNS), focusing on their role in anxiety disorders. The involvement of miRNAs in CNS functions (such as neurogenesis, neurite outgrowth, synaptogenesis and synaptic and neural plasticity) and their intricate regulatory role under stressful conditions strongly support their importance in the aetiology of anxiety disorders. Furthermore, miRNAs could provide new avenues for the development of therapeutic targets in anxiety disorders.

## Background

### Anxiety Disorders

Anxiety disorders are a heterogeneous group of disorders that include acute stress disorder, agoraphobia (with or without a history of panic disorder), generalised anxiety disorder (GAD), obsessive-compulsive disorder (OCD), panic disorder (PD) (with or without agoraphobia), phobias (including social anxiety disorder) and posttraumatic stress disorder (PTSD) [1]. These disorders cause significant distress and functional impairments, and collectively have an estimated lifetime prevalence of up to 25 % [2]. The global prevalence of current anxiety disorders has been estimated at 7.3 % (4.8–10.9 %) and ranges from 5.3 % (3.5–8.1 %) in African cultures to 10.4 % (7.0–15.5 %) in Euro/Anglo cultures [3].

### Neurobiology of Anxiety

Anxiety is an evolutionary trait that provides a coping mechanism in dangerous environmental situations and is associated with emotional processes and cognitive functions, such as learning and memory. These cognitive functions are underpinned by several neural substrates and neurotransmitter pathways that are characterised by a high degree of plasticity [4]. Functional magnetic resonance imaging (fMRI) studies have revealed increased baseline activity in the parahippocampal gyrus and the cingulate cortex [5] and increased brain activity in the amygdala, parahippocampal gyrus and frontal cortex in response to anxiety-inducing stimuli [6]. These findings suggest an important role for the forebrain, as a site of increased excitatory neurotransmission, in the anxiety disorders.

An important physiological hallmark of anxiety is excessive excitatory neurotransmission [7]. The hypothalamic–pituitary–adrenal (HPA) axis is an integral component in the neuroendocrine response to acute stress. Corticotropin-releasing hormone (CRH) regulates the stress-induced activation of the HPA axis and mediates autonomic and behavioural changes associated with anxiety disorders [8]. CRH and vasopressin are secreted in response to stress by the hypothalamus. These neuropeptides are secreted into the portal vessels and stimulate the anterior pituitary to synthesise and release adrenocorticotropin hormone (ACTH), which in turn leads to the release of glucocorticoids (GCs) by the adrenal cortex. GCs help to control the processes of adaptation and recovery due to the role they play in the restoration of biological homeostasis [9, 10]. Studies have shown a link between elevated cortisol and both chronic stress and depression [11].

### Current and Future Treatments for Anxiety Disorders

Different treatment options are available for anxiety disorders, including cognitive behavioural therapies [12] and pharmacological treatment options. Historically, tricyclic antidepressants (TCAs) have been widely used in the treatment of anxiety disorders and have demonstrated similar efficacy to the SSRIs for panic disorder and generalised anxiety disorder (GAD). However, the less tolerable side effect profile of TCAs and concerns about safety (including problematic anticholinergic and antiadrenergic effects), as is true of the monoamine oxidase inhibitors (MAOIs) (side effects include insomnia, sedation, hypotension, sexual dysfunction, hypomania, weight gain/oedema, hypertensive episode and myoclonic jerking), do not make them first-choice drugs. The selective serotonin re-uptake inhibitors (SSRIs) (e.g. fluoxetine, paroxetine, sertraline) are considered to be first-line pharmacological agents for all of the anxiety disorders, with evidence from multiple, randomised, placebo-controlled trials supporting their efficacy and safety [13]. Serotonin norepinephrine reuptake inhibitors (SNRIs) (e.g. venlafaxine) are also emerging as first-line medications for some of the anxiety disorders, most notably GAD. SSRIs and SNRIs block serotonin (5-HT) and norepinephrine re-uptake after release from neurons, respectively, resulting in their increased availability at the synapse, increased potency of neurotransmission and further downstream effects on other neurotransmitters [14]. The slower therapeutic onset of SSRIs (2 to 4 weeks) is associated with gradual changes in both brain structure and function [15]. Animal investigations have shown that proliferation of new neurons in the hippocampus contributes to the behavioural effects of SSRIs [16, 17], while modifications in plasticity could be a putative mechanism whereby these drugs counteract hyperresponsivity to stress in anxiety disorders [18]. Although more effective than some of the older treatments, SSRIs have limited efficacy in a subset of patients and the side effects together with the delayed onset of action influence compliance [19].

Another class of agents, the benzodiazepines, are potent and fast-acting but are not a recommended first choice for anxiety in view of their potential for physiological dependence and their propensity for troublesome adverse effects (e.g. sedation and cognitive impairment), and rebound anxiety (upon discontinuation) [20]. Benzodiazepines enhance the inhibitory effects of γ-aminobutyric acid (GABA), the main inhibitory neurotransmitter in the brain, through their action on GABA_A_ receptors [21]. Similar to the benzodiazepines in terms of their effects on inhibitory/excitatory neurotransmission, the anticonvulsants pregabalin and gabapentin demonstrate superior efficacy to placebo in GAD and social anxiety disorder. However, these two drugs do not have the same abuse and dependence potential as the benzodiazepines.

The majority of patients with anxiety disorders do not respond completely to an initial treatment trial, necessitating a switch in drug or the addition of a second medication. Furthermore, the side effect profiles of most anti-anxiety treatments significantly hamper patient compliance. There are consequently efforts under way to identify novel pharmacological targets, including investigation of neuropeptide Y (NPY) receptor agonists, vasopressin (V1B) antagonists, NMDA receptor antagonists, and pharmacological modulators of learning and memory. One such agent is d-cycloserine (DCS). DCS administration (before or immediately after extinction training) has been shown to be an effective therapeutic target in facilitating extinction learning in anxiety disorders, such as specific and social phobias, OCD and PTSD [22–24]. DCS is a partial *N*-methyl-d-aspartate receptor (NMDAR) agonist at the glycine site on the NMDAR1 receptor subunit. NMDARs are critical for the neural plasticity underlying learning under normal conditions [25]. Activation of the NMDARs requires the binding of both glutamate and the co-agonist glycine for efficient opening of the calcium channel. Upon opening of the channel, intracellular calcium concentrations increase which activates signal transduction pathways critical to the plasticity underlying fear extinction [26]. One of the mechanisms underpinning the formation of emotional (including fear) memories is the interaction between DCS and other fear extinction enhancers (histone deacetylase inhibitors) with brain-derived neurotrophic factor (BDNF) and tropomyosin-related kinase B receptors in the amygdala, hippocampus and prefrontal cortex [27]. Other potential treatment approaches include glucocorticoid receptor, corticotropin-releasing factor and norepinephrine signalling modulators that may alter stress responses. Glucocorticoid receptor modulators and modulators of glutamate signalling (positive allosteric modulators of glutamate receptors, glycine transporter inhibitors and glycine agonists also have therapeutic potential) are putative cognitive enhancers that target mechanisms of conditioned fear extinction [28].

### Genetics of Anxiety

Numerous factors play a role in the aetiology of anxiety disorders, such as cognitive and physiological factors, genetic heterogeneity as well as epigenetic or regulatory changes. Several animal and human studies have investigated the role of molecular mechanisms in anxiety disorders. Results from twin studies that have investigated the heritability of GAD, PD, phobias and PTSD point to a complex interplay of genetics and the environment [29]. More recently, genetic screening in complex disorders has been extended to the identification of rare risk alleles, copy number variations (CNVs) and regulatory elements such as miRNAs. Genetic variation in regulatory gene regions may play a major role in phenotypic diversity [30, 31], whereby minor variations in gene regulation have the potential to alter gene dosage and contribute to genetic susceptibility to disease. In this regard, it is important that regulatory elements acting in the brain be more thoroughly studied in anxiety disorders.

### MicroRNAs (miRNAs)

MicroRNAs (miRNAs) are a class of small, noncoding RNAs that have recently drawn interest as epigenetic modulators of gene expression in psychiatric disorders [32]. In 1993, the first miRNA, lin-4, was discovered in *Caenorhabditis elegans* through genetic screening for deficiencies in the temporal control of postembryonic development [33]. However, it was only in 2001 that the role of miRNAs as a new layer of gene regulation was finally appreciated [34–36].

MiRNAs are single-stranded RNA species approximately 22 nucleotides (nt) long that form part of a large class of small, noncoding RNAs. miRBase is the major online repository for all miRNA sequences and annotation. The most recent version of the database, release 19, contains 21,264 hairpin precursor miRNAs entries expressing 25,141 mature miRNA products, in 193 species [37]. Between 1 % and 5 % of mammalian genes are comprised of miRNAs [38], making them one of the most abundant classes of regulators in the genome [39]. Half of all the miRNAs are expressed from non-protein coding transcripts and the other half from intronic regions of protein-coding genes [40]. MiRNAs are evolutionarily conserved and are involved in numerous intricate processes including the stress response [41]. They are of particular importance in brain functioning and are involved in learning and memory processes [42] as well as synaptic plasticity [43]. Certain miRNAs are ubiquitously expressed (e.g. let-7b, miR17-5p and miR21) [44] while others have an expression pattern dependent on the specific cell type or developmental stage [45] (e.g. brain and spinal cord-specific miR34a [44], and miR409-3p in brain development in mice [46]).

**Fig. 1:**
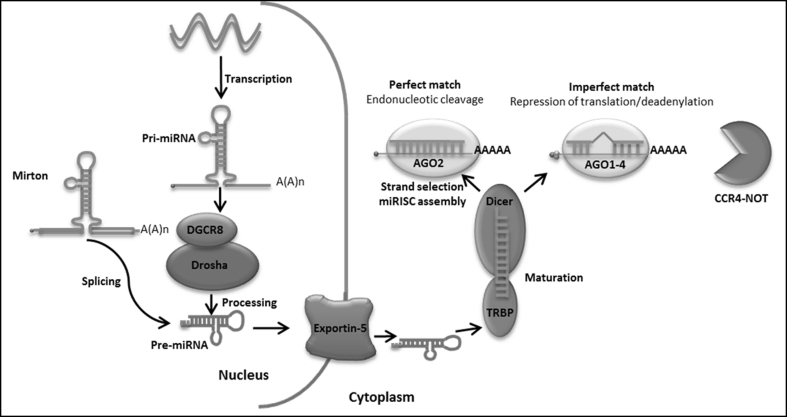
Figure depicting the production of mature miRNAs. MicroRNAs (miRNAs) are encoded in the genome, their genes usually transcribed by RNA polymerase II. The transcripts undergo splicing and polyadenylation. The pri-miRNA is processed in the nucleus by the Drosha RNaseIII enzyme and the DGCR8 protein, producing the pre-miRNA. The pre-miRNA is exported to the cytoplasm by exportin-5 where Dicer and the TRBP cleave the pre-miRNA to yield a miRNA duplex (about 22-bp long). One strand is selected to function as a mature miRNA, the other strand is usually degraded. Mature miRNAs are then incorporated in a miRNA-induced silencing complex (miRISC) that recognises and binds to the 3′ UTR of the target mRNA and represses translation (AGO-argonaute) [48]

The production of mature miRNAs is a complex process; the primary transcript miRNAs (pri-miRNAs) are cleaved by the ribonuclease III (Drosha) enzymes and the DiGeorge syndrome critical region gene 8 protein (DGCR8) in the nucleus. This cleavage produces a precursor miRNA (pre-miRNA) approximately 70–100 nt in length that is actively transported to the cytoplasm by exportin 5. In the cytoplasm, the pre-miR is cleaved by another RNaseIII enzyme, Dicer, and the trans-activation responsive (TAR) RNA binding protein (TRBP) to generate double-stranded miRNAs approximately 22 nt in length. Thereafter, a helicase unwinds the dsRNA of the miRNA and one of the strands is degraded while the other (known as the guide strand) functions as the mature miRNA. The mature miRNA is incorporated into a miRNA-induced silencing complex (miRISC), a complex of proteins that target mRNAs based on sequence complementarity mostly in the 3′ untranslated regions (UTRs) [47]. In the case of perfect complementarity between the miRNA and target mRNA, the target RNA is degraded. In the absence of perfect complementarity, the target is not cleaved but is deadenylated which leads to decapping and subsequent exonucleolytic digestion or translational repression (through a different mechanism at each translational step, namely initiation, elongation and termination) [48] (Fig. 1) [9]. It is important to note that not all mRNA targets are directly targeted by miRNAs via binding to the 3′ UTR of the mRNA. Indirect targets form part of a miRNA-mediated regulatory pathway but do not possess structural affinity for miRNAs. However, the expression of these targets is indirectly affected by another target of the miRNA [49]. Parker and Wen have also shown that indirect targets have a delayed response in expression changes over time compared to direct targets (as described for miR-124) [50]. It is clear that miRNAs do not simply turn genes on and off, but form part of an interconnected regulatory network that fine-tunes the expression levels of target genes [51]. Variations in target sites could thus result in altered gene expression patterns and ultimately contribute to disease susceptibility [52].

### MiRNA Targets

Approximately 20–30 % of all genes are regulated by at least one miRNA [38, 53, 54]. However, computational analysis suggests that a single miRNA can target hundreds of genes and that one gene can be targeted by more than one miRNA [55]. Although the 3′ UTR of mRNAs is a typical target site of miRNAs, target sites in the coding region have also been documented [56, 57]. Nucleotides 2–7 of the miRNA sequence are known as the seed region and are the most critical region for target recognition [58]. MiRNA-mediated regulation of mRNAs is complicated by the fact that miRNAs are prone to tissue-specific RNA editing. RNA editing is a posttranscriptional mechanism whereby some RNA molecules are changed to contain bases not originally encoded in the genome (via nucleotide insertion, deletion or modification). Such events can lead to altered properties of miRNAs and alternative mRNA–miRNA interactions [59].

In order to gain insight into miRNAs and their functions, it is essential to identify their mRNA targets. This step has proven to be computationally challenging. Although great advances have been made in the field of miRNA target prediction, with the development of various target-predicting software [60], their false-positive rates of target prediction range between 24 % and 70 % [61–63]. These high rates emphasise the importance of experimental strategies to validate predicted targets in an endeavour to identify genuine miRNA targets and miRNA function [64]. For more detail on in vitro and in vivo experimental strategies for miRNA target identification, refer to Thomson et al. [64], Schratt et al. [43], Pasquinelli et al. [65] and Karres et al. [66].

### MiRNAs in the Central Nervous System (CNS) and Synaptic Plasticity

Both acute and chronic stress are associated with the development of anxiety disorders through intricate mechanisms related to neural plasticity [67]. Optimal functioning of the CNS requires precise and rapid changes in gene regulation; posttranscriptional regulation by miRNAs could represent one of the ways in which this is achieved [39]. Numerous miRNAs are abundantly expressed in fully differentiated neurons of the mature brain [48]. Recent studies suggest a crucial role for miRNAs in regulating various neurobiological processes, including neurogenesis, neurite outgrowth, synaptogenesis and synaptic and neural plasticity [68]. Many putative miRNA targets are involved in neural development; these include mRNAs that encode proteins involved in the maintenance of neuronal function, plasticity of neural networks and specific neurodevelopmental and neurodegenerative diseases [69]. Studies have also shown that miRNAs are altered by stress, glucocorticoids and mood stabilisers [32], suggesting that miRNAs could be vital in the aetiology of anxiety disorders. Hunsberger suggested that miRNAs could be differentially expressed in patients with various psychiatric disorders, indicating that miRNAs may have the potential to broaden our understanding of the pathophysiology and therapeutics of anxiety disorders [32].

The role of miRNAs as translational regulators also implicates these small RNAs as mediators of long-term plasticity. Hansen et al. investigated the functions of miRNAs in synaptic plasticity and discovered that miR-134 modulates synaptic plasticity in the rat hippocampus [70]. Further studies suggest that miRNAs are involved in the critical phases of memory formation via synaptic tagging to ensure synaptic input specificity [71–73]. A model for miRNA-mediated effects at the synapse has been proposed [74], in which miRNAs could exert an influence on transcripts with stimulation-dependent translation, such as the ionotropic glutamate receptors stimulated by glutamate [48, 74].

Mature miRNAs have been shown to play a vital role in early mammalian development. Knockout studies in zebrafish deficient in the Dicer protein (and consequently lacking functional miRNAs) indicate an important function for miRNAs in the formation of the embryonic neural plate and its transformation into the neural tube [75]. Studies in mice suggest that miR-9 and miR-124a are involved in neural lineage differentiation in embryonic stem (ES) cell-derived cultures [76]. In fact, expression of miR-124a, even in nonneuronal cells, induces an overall neuronal gene-expression pattern [77, 78]. Upon introduction into HeLa cells, miR-124a induces a neuronal-like expression profile by decreasing the levels of numerous nonneuronal transcripts. Alternatively, repressor element-1 (RE-1) silencing transcription factor (transcriptional repressor) inhibits the expression of neuronal genes, including that of miR-124a, in nonneuronal cells [79].

MiRNA-132 was identified as one of the most highly inducible CREB targets in a genome-wide study by Vo et al. where they screened for cAMP-response element binding (CREB) protein targets that directly regulate neuronal plasticity. CREB protein acetylates histones, giving a specific tag for transcriptional activation; it also binds to phosphorylated CREB and improves its transcriptional activity toward cAMP-responsive genes. In addition, the authors showed that miR-132 expression in cortical neurons induced neurite outgrowth and, conversely, that its inhibition reduced neuronal outgrowth [80]. Their research suggests that miR-132 decreases the levels of the GTPase-activating protein (p250GAP), a protein proposed to regulate neuronal differentiation, and subsequently neuronal morphogenesis [80–82]. The role that this miRNA plays in neuronal plasticity also suggests a role for miR-132 in regulating expression changes associated with anxiety disorders.

Several researchers have also set out to identify specific miRNAs and their targets responsible for regulating synaptic function. Schratt et al. investigated the brain-specific miRNA regulation of dendritic spine development in rats. Most dendritic spines contain a postsynaptic density (PSD) (comprising of a complex matrix of postsynaptic receptors, cytoskeletal proteins and signalling molecules) and are involved in postsynaptic signalling and plasticity [83]. They found that miR-134, a brain-specific miRNA, modulates synaptic plasticity in the rat hippocampus. This miRNA has a negative effect on the size of dendritic spines in rat hippocampal neurons by directly targeting *LIMK1* (Lim-domain containing protein kinase 1) [43]. *LIMK1* is a potent regulator of cofilin and actin dynamics and plays a crucial role in the morphogenesis of dendritic spines and brain function [84]. Further investigations of miRNAs that regulate dendritic spine morphology and synaptic plasticity will provide valuable insight into the intricate processes involved in learning and memory.

### MiRNAs in Anxiety as Described in Animal Models

The HPA axis plays a vital role in regulating the normal response to stress; malfunctioning of this system underlies susceptibility to certain anxiety disorders [10]. Precise mechanisms for this increased susceptibility has however not been fully elucidated. miRNAs are abundantly expressed throughout the brain, where they perform important regulatory functions in the CNS [48, 51]. This suggests a role for miRNAs in stress response regulation. Uchida et al. established and characterised an animal model of vulnerability to repeated stress [85]. Previous studies have shown that Fischer 344 (F344) rats constitute a stress-hyperresponsive rat strain that is more vulnerable to repeated restraint stress (RRS) compared to other strains such as Sprague–Dawley (SD) rats. Uchida and colleagues first investigated neuroendocrine and biochemical responses to RRS and found lower levels of glucocorticoid receptor (GR) protein expression in the paraventricular nucleus (PVN) in F344 rats compared to control SD rats. They focused on translational repression by miRNAs to help explain the observed aberrant translation of GR. They established that miR-18a inhibited translation of GR mRNA (in cultured neuronal cells) and that higher expression levels of miR-18a were present in F344 rats compared with SD rats in the PVN. In vitro experiments confirmed the results for miR-18a and also established a similar role for miR-124 [86]. MiR-18 and miR-124a reduced the levels of GR protein and decreased GR-mediated events. MiR-18 is widely expressed throughout the body, whereas miR-124a expression is restricted to the brain [86]. Down-regulation of GR translation via miR-18a may be an important susceptibility mechanism for stress-related disorders [85], and F344 rats could therefore be a useful animal model for studying vulnerability to repeated stress.

Subsequent work by Uchida et al. focused on the effects of maternal separation and early life adversity on the behavioural response to RRS as well as vulnerability to chronic stress in adult rats [87]. Maternally separated rats showed increased expression of repressor element-1 silencing transcription factor 4 (REST4), a neuron-specific splicing variant of the transcriptional repressor REST. REST regulates certain brain-enriched miRNAs postulated to be associated with neuronal functions such as brain development and plasticity [74, 79, 80, 88]. The maternally separated rats also showed a marked increase in a variety of REST target gene mRNAs and miRNAs in the medial prefrontal cortex (mPFC). The expression of pre-mir132, -124-1, -9-1, -9-3, -212 and -29a as well as the mature miR132, -124, -9 and -29a were found to be significantly up-regulated in maternally separated rats compared to control rats. Interestingly, mir-132, -124-1, -9-1, -9-3, -212 and -29a all possess a repressor element-1 (RE-1) site within 50 kb of their promoter regions [88]. The authors hypothesised that the differential expression of mRNAs and miRNAs of genes that contain RE-1 might be due to alterations in RE-1-mediated gene transcription in the mPFC of maternally separated rats secondary to altered REST4 expression. Indeed, results indicated an increased level of expression of genes and miRNAs possibly regulated by REST4, such as glutamate receptor subunit (*Glur2*), calcium/calmodulin-dependent protein kinase II (*CamKIIα*) and adenylate cyclase 5 (*Adcy5*) as well as precursors for mir132, -124 and -212. These results suggest a role for an REST4-mediated gene network and specific miRNAs acting in the mPFC. This study provides additional insights into factors that could influence susceptibility to developing mood and anxiety disorders in adulthood following exposure to early life stress. [87].

Meerson et al. predicted that miRNAs mediate stress response regulation through alternative splicing. They studied expression profiles of miRNAs in the hippocampus CA1 region and the central amygdala in both acute and chronically stressed rats. They found that both acute and chronic immobilisation stress induced distinct miRNA expression profiles in these two stress-responsive brain regions. MiR-134 and miR-183 were up-regulated in the amygdala following acute stress. MiR-134 was down-regulated in the amygdala and hippocampus under chronic stress conditions in both the amygdala and CA1. These two miRNAs were further investigated as they shared numerous common predicted mRNA targets that were known mediators of neuronal stress reactions, including the Serine/Arginine-rich splicing factor 2 (SC35). SC35 is up-regulated in response to stress, promoting the alternative splicing of acetylcholinesterase (AChE) from its synapse-associated isoform (AChE-S) to the rare soluble form of the protein (AChE-R). MiR-183-mediated suppression of SC35 was confirmed in cultured cells. This alternative splicing of AChE affects the local and temporal regulation of cholinergic neurotransmission. The authors were able to demonstrate that stress altered the expression levels of miR-183 and miR-134. Through regulating splicing factors and their targets, these miRNAs were able to modify both alternative splicing and cholinergic neurotransmission under stress conditions in the brain, providing a link between the molecular and physiological responses of different brain regions to psychological stress [89].

The functional role of miRNAs in regulating stress responses were investigated by Haramati et al.; by inactivating the Dicer gene (a key enzyme in miRNA synthesis pathway), they were able to inactivate miRNA processing in the central amygdala [90]. A sharp increase in anxiety-like behaviour was evident in mice lacking Dicer (and thus also mature miRNAs) in their amygdala. In addition, acute stress in wild-type mice induced differential expression of numerous miRNAs in the amygdala. MiR-34c, one of the prominent stress-induced miRNAs, was further investigated and found to be strongly up-regulated by exposure to stress, resulting in reduced symptoms of anxiety in normal mice. Interestingly, corticotrophin-releasing factor receptor type 1 (CRFR1) mRNA is one of the targets of miR-34c. The authors showed that miR-34c elicits its effect on the amygdala by targeting an evolutionarily conserved region in the 3′ UTR of CRFR1 mRNA. The authors postulated that miR-34c down-regulates stress-related proteins like CRFR1 and assists in the stress recovery process of these mice. In effect, such miRNAs and their targets may unveil new targets for the treatment of stress-related disorders [90].

By 2008, it was established that miRNAs play an important regulatory role in neuronal development; however, the mechanism of regulation of miRNA expression had not been elucidated. Parsons et al. investigated differential miRNA expression in one tissue of different inbred mouse strains to gain more insight into miRNA expression regulation. By studying differential miRNA expression in the hippocampus of four common inbred mouse strains (A/J, BALB/cJ, C57BL/6J and DBA/2J) prone to anxiety-like behaviour, they identified 11 differentially expressed miRNAs. The expression of miR-34a, miR-323, miR-378 and miR-451 correlated with behavioural measurements of exploration on the elevated plus maze task (indicative of anxiety levels), with less anxious animals displaying more explorative behaviour. MiR-34c and miR-323 expressions correlated with anxiety (less explorative behaviour) on the elevated plus maze task and expression of miR-34c, miR-323, miR-378 and miR-451 correlated with tests of learning and memory [91]. While a role for miRNAs in synaptic development had previously been proposed [42], this study was one of the first to demonstrate involvement of miRNAs in anxiety, learning and memory.

Acute and repeated stress affects neural activity in different brain regions [92]; short-term changes in neural transmission and gene regulation [93–95] and longer term changes in structural modification [96–98] have, in particular, been documented. It is thus plausible that miRNAs may be involved in these processes. In a recent study investigating the effects of single or repeated exposures to restraint stress on miRNAs in the frontal cortex of CD1 mice, a marked increase in the expression levels of various miRNAs after acute stress was found, while only minor changes were observed after repeated restraint. The authors hypothesised that acute stress rapidly modulates miRNAs, but that these effects are only transient. Northern blot analysis confirmed that after acute restraint an increase in let-7a, miR-9 and miR 26-a/b was observed. These changes were found to be region specific, present in the frontal cortex but not in the hippocampus, providing evidence that miRNAs in the frontal cortex are involved in the process of translating stressful events to alterations in protein expression [99].

### MiRNAs in Anxiety as Described in Human Studies

The role that miRNAs play in synaptic plasticity and neuronal differentiation suggests that miRNAs may be involved in the aetiology of numerous psychiatric disorders. Various miRNA expression studies have been conducted in schizophrenia patients (post-mortem brain samples) [100–102], autism spectrum disorders [103, 104], Rett syndrome [105] and substance abuse disorders [106]. To date, there have been few studies of miRNAs in anxiety disorders.

Muiños-Gimeno et al. selected a panel of SNPs (712 SNPs that covered 325 miRNA regions) to use in association studies of panic disorder [107]. Their analysis revealed that the SNP coverage in miRNA regions is much lower than the rest of the genome. None of these SNPs were located within a mature miRNA sequence, which is in line with the reported negative selection at miRNAs and miRNA target sites at 3′ UTRs [108]. This lower SNP density was confirmed by a study that re-sequenced 117 miRNAs in four different human reference populations [109]. It is thus evident that mutations in miRNA binding sites are likely to be deleterious and could have severe phenotypic implications. Re-sequencing of 3′ UTRs and miRNAs in patients and controls might cast more light on the role of miRNA-mediated regulation in the susceptibility to anxiety disorders [109].

In 2011, Muiños-Gimeno et al. [107] investigated the functional role of miRNAs in panic disorder (PD) in a Spanish cohort of patients with PD. They examined 712 single-nucleotide polymorphisms (SNPs) that tagged 325 human miRNA regions. Two SNPs found to be significantly associated with panic disorder, rs6502892 and rs11763020, were also found to tag miRNAs miR-22 and miR-339, respectively. MiRNA-22 was shown to regulate four candidate genes, namely brain-derived neurotrophic factor *BDNF*, serotonin 5-HT2C receptor (*HTR2C*), monoamine oxidase A (*MAO-A*) and the regulator of G-protein signalling 2 gene (*RGS2*). Target predicting software proposed adenosine receptor A2a (*ADORA2A*), *BDNF*, corticotropin-releasing hormone receptor 2 (*CRHR2*) and sodium-dependent noradrenaline transporter (*SLC6A2*) as possible targets of miR-339. In addition, they found SNPs associated with PD sub-phenotypes (PD with and without agoraphobia) that tagged miR-138-2, miR-148a, miR-488 and miR-491. Functional studies indicated that miR-138-2, miR-148a, and miR-488 repressed the expression of certain candidate genes for PD in the region of 30 % to 60 %, including gamma-aminobutyric acid A receptor, alpha 6 (*GABRA6*), cholecystokinin B receptor (*CCKBR*) and proopiomelanocortin preproprotein (*POMC*), respectively [107]. Following transfection with miR-22 and miR-488, neuroblastoma cells showed altered expression of a subset of potential target genes for these miRNAs and genes that might affect physiological pathways related to anxiety. An association between rs73531, which tagged the intergenic miR-148a, and age at onset (AAO) (*p* = 0.0007) was observed. The average AAO was 23 years for the *GG* homozygotes and 30 years for the *AG* heterozygotes and *AA* homozygotes [107].

Neurotrophin-3 growth factor receptor (*NTRK3*) was also investigated as a candidate susceptibility factor in PD and obsessive-compulsive disorder (OCD). After re-sequencing the 3′ UTRs in two different isoforms of *NTRK3* in PD and OCD patients, they found that in the truncated isoform of *NTRK3* (located in a functional target site for miR-485-3p) the *C* allele of rs28521337 was significantly associated with the hoarding phenotype of OCD. Additionally, they identified two new rare variants, ss102661458 (located in a functional target site for miR-765) and ss102661460 (located in a functional target sites for miR-509 and miR-128), in the 3′ UTR of *NTRK3*, present in one chromosome of a PD patient [52]. MiR-128 is a brain-enriched miRNA that is involved in synaptic processing and neuronal differentiation and miR-509 shares the target site of miR-128, its expression is restricted to the testis [110], suggesting tissue-dependent regulation of NTRK3 at this site. These two variants resulted in the recovery of gene expression by significantly altering the miRNA-mediated regulation of *NTRK3*. Their data provides evidence that miRNAs play a key role in posttranscriptional regulation, in this case allele-specific miRNA regulation of *NTRK3* in anxiety disorders [52].

A cross-species approach is another interesting method that has been used to study anxiety and to identify genes that regulate anxiety-like behaviour. This approach has enabled researchers to identify a SNP (rs817782) in the 3′ UTR of the aminolevulinate dehydratase gene (*ALAD*) that was shown to be associated with social phobia [111]. The rare *A* allele of rs817782 generated a putative target site for both miR-211 and miR-204 within the *ALAD* 3′ UTR, as predicted by a miRNA target prediction program (http://www.patrocles.org) [111]. The authors previously found that *ALAD* was expressed at a higher level in the hippocampus and periaqueductal grey of six inbred anxious mouse strains. These two brain regions together are part of the abnormally sensitive fear network that patients with PD suffer from. However, a direct link between this functional *ALAD* SNP, the putative miRNA target sites (for miR-211 and miR-204) and PD has yet to be established [112].

### MiRNAs and Pharmacotherapies for Anxiety Disorders

The serotonin transporter (SERT) is an important neurotransmitter in the CNS that ensures the reuptake of serotonin at the synaptic cleft and regulates serotonin levels in the brain. Defective serotonergic neurotransmission has been associated with anxiety, OCD, depression and suicidal behaviour [113, 114]. SERT is also a pharmacological target of selective SSRI antidepressants [115], one of the very effective treatments for various anxiety disorders. A study by Baudry et al. found that SERT is a target of miR-16. After chronically treating mice with the SSRI fluoxetine (Prozac), there was an increase in miR-16 levels in serotonergic raphe nuclei that resulted in reduced SERT expression [116]. These studies clearly confirm the important role of miRNAs in the pathophysiology of anxiety disorders. Furthermore, miRNAs present a novel therapeutic strategy as targets for anxiolytic drugs. Since miRNAs play an essential role in regulating numerous stress response pathways, it is imperative that miRNAs be evaluated as potential drug targets for anxiety disorders.

Zhou et al. conducted one of the first studies that demonstrated that miRNAs and their effectors are targets of pharmacotherapeutic drugs. Lithium and valproate (VPA) have been found to be effective in treating bipolar disorder (BPD). Although not routinely used in the anxiety disorder setting, valproate in particular may be a useful adjunct in treatment-refractory anxiety disorder patients as well as in those patients with a comorbid bipolar disorder and might enhance exposure-based cognitive therapy for anxiety disorders and PTSD [117]. Zhou et al. found fluctuating levels of various hippocampal miRNAs following chronic treatment with mood stabilisers, lithium and VPA. The miRNAs that they were able to confirm were let-7b, let-7c, miR-24a, miR-30c, miR-34a, miR-128a, miR-144 and miR-221. The predicted effectors of these miRNAs are involved in neurogenesis, neurite outgrowth and signalling of extracellular signal-regulated kinase (ERK), phosphatase and tensin homologue deleted from chromosome 10 (PTEN) and Wnt/β-catenin pathways [68]. Treatment with mood stabilisers such as lithium and VPA has been found to increase the expression of genes encoding dipeptidyl-peptidase 10, metabotropic glutamate receptor 7 (*GRM7*) and thyroid hormone receptor β in vivo [68]. Several of these effector-coding genes have previously been described as candidates for susceptibility to the development of BPD. The authors went on to investigate the effects of lithium and VPA on the expression of miRNAs and their effectors in primary cultures. Primary cultures that received treatments of lithium or VPA showed lowered levels of miR-34a and elevated levels of GRM7 (a predicted effector of miR-34a). In addition, treatment with a miR-34a precursor decreased GRM7 levels and treatment with a miR-34a inhibitor increased GRM7 levels. These results confirm that endogenous miR-34a regulates the levels of GRM7, which may contribute to the therapeutic effects of lithium and VPA on GRM7 [68]. Valproate has been shown to be effective, particularly as an augmentation strategy, for a number of anxiety disorders, including PTSD, panic disorder, GAD and SAD [68].

Table 1 provides all the miRNAs included in the review that have been implicated in the aetiology of anxiety disorders.

**Table 1:** Summary of microRNAs that are possibly involved in anxiety disorders

MiRNA	Involvement with anxiety disorders	Species	Reference
Let-7a-1	Up-regulated expression in the frontal cortex following acute stress	*Mus musculus*	Rinaldi et al. [99]
	Down-regulated in amygdala after acute and chronic stress	*Rattus norvegicus*	Meerson et al. [89]
Let-7b	Increased expression in the hippocampus due to treatment with lithium and sodium valproate	*Rattus norvegicus*	Zhou et al. [68]
Let-7c	Decreased expression in the hippocampus due to treatment with lithium and sodium valproate	*Rattus norvegicus*	Zhou et al. [68]
miR-1	Up-regulated in amygdala under chronic stress and down-regulated in the hippocampus under acute stress	*Rattus norvegicus*	Meerson et al. [89]
miR-9	Involved in neural lineage differentiation in ESCs	*Mus musculus* and in vitro cell line	Krichevsky et al. [76]
	Up-regulated expression in the frontal cortex following acute stress	*Mus musculus*	Rinaldi et al. [99]
	Up-regulated expression in the medial pre-frontal cortex following maternal separation	*Rattus norvegicus*	Uchida et al. [87]
miR-9-1	Pre-miRNA up-regulated expression in the medial pre-frontal cortex following maternal separation	*Rattus norvegicus*	Uchida et al. [87]
	Down-regulated in CA1 region of hippocampus under acute or chronic stress		Meerson et al. [89]
miR-9-3	Pre-miRNA up-regulated expression in the medial pre-frontal cortex following maternal separation	*Rattus norvegicus*	Uchida et al. [87]
miR-17-5p	Up-regulated in the hippocampus CA1 region under chronic stress	*Rattus norvegicus*	Meerson et al. [89]
	Controls neuronal development and differentiation	In vitro cell line	Hebert et al. [118]
miR-18a	Possible repressor of the glucocorticoid receptor gene in the hypothalamic paraventricular nucleus regulating stress responses	*Rattus norvegicus*	Uchida et al. [85], Vreugdenhil et al. [86]
miR-21	Involved in the control of glial cell differentiation	In vitro cell line	Chan et al. [119]
miR-22	Associated with panic disorder—repression of *RGS2*, *BDNF*, *HTR2C* and *MAOA*	*Homo sapiens*	Muiños-Gimeno et al. [107]
miR-24a	Decreased expression in the hippocampus due to treatment with lithium and sodium valproate	*Rattus norvegicus*	Zhou et al. [68]
miR-26a/b	Up-regulated expression in the frontal cortex following acute stress	*Mus musculus*	Rinaldi et al. [99]
miR-29a	Up-regulated expression in the medial pre-frontal cortex following maternal separation	*Rattus norvegicus*	Uchida et al. [87]
miR-30c	Decreased expression in the hippocampus due to treatment with lithium and sodium valproate	*Rattus norvegicus*	Zhou et al. [68]
miR-34a	Correlation between differential expression of this miRNA and behavioural measures for exploration on the elevated plus maze task	*Mus musculus*	Parsons et al. [91]
	Decreased expression in the hippocampus due to treatment with lithium and sodium valproate	*Rattus norvegicus*	Zhou et al. [68]
miR-34c	Correlation between differential expression of this miRNA and behavioural measures for anxiety in mice. Up-regulated by exposure to stress	*Mus musculus*	Parsons et al. [91]
			Haramati et al. [90]
miR-124	Up-regulated expression in the medial pre-frontal cortex following maternal separation	*Rattus norvegicus*	Uchida et al. [87]
miR-124-1	Pre-miRNA up-regulated expression in the medial pre-frontal cortex following maternal separation	*Rattus norvegicus*	Uchida et al. [87]
	Down-regulated in the hippocampus under acute stress. Controls neuronal development and differentiation		Meerson et al. [89]
			Hebert et al. [118]
miR-124a	Involved in neural lineage differentiation in ESCs	*Mus musculus* and in vitro cell line	Krichevsky et al. [76]; Lim et al. [77]; Makeyev et al. [78]
	Down-regulates glucocorticoid receptor	*Rattus norvegicus*	Vreugdenhil et al. [86]
miR-128	Association of an allelic variant in the target site for miR-128 in *NTRK3* (*ss102661458*) with panic disorder—reduction of *NTRK3* repression	*Homo sapiens*	Muiños-Gimeno et al. [52]
miR-128a	Decreased expression in the hippocampus due to treatment with lithium and sodium valproate	*Rattus norvegicus*	Zhou et al. [68]
miR-128b	Regulates formation of fear-extinction memory in the infralimbic pre-frontal cortex	*Mus musculus*	Lin et al. [120]
miR-132	One of the most highly inducible CREB targets, plays a role in neurite outgrowth and neuronal plasticity	In vitro neural cell line	Vo et al. [80]
	Up-regulated expression in the medial pre-frontal cortex following maternal separation	*Rattus norvegicus*	Uchida et al. [87]
	Pre-miRNA up-regulated expression in the medial pre-frontal cortex following maternal separation		
miR-134	Modulates synaptic plasticity in hippocampus	*Rattus norvegicus*	Hansen et al. [70]
	Up-regulated expression in the central amygdala and hippocampus after acute stress	*Rattus norvegicus*	Meerson et al. [89]
	Down-regulated expression in the central amygdala and hippocampus after chronic stress	*Rattus norvegicus*	Meerson et al. [89]
miR-138-2	Associated with age at onset in panic disorder—repression of *GABRA6*	*Homo sapiens*	Muiños-Gimeno et al. [107]
miR-144	Decreased expression in the hippocampus due to treatment with lithium and sodium valproate	*Rattus norvegicus*	Zhou et al. [68]
miR-148a	Associated with age at onset in panic disorder—repression of *CCKBR*	*Homo sapiens*	Muiños-Gimeno et al. [107]
miR-183	Up-regulated expression in the central amygdala following acute stress	*Rattus norvegicus*	Meerson et al. [89]
miR-204	Association of an allelic variant in the 3′ UTR of *ALAD* with SP	*Homo sapiens*	Donner et al. [111]
miR-208	Up-regulated in CA1 region of hippocampus under acute or chronic stress	*Rattus norvegicus*	Meerson et al. [89]
miR-211	Association of an allelic variant in the 3′ UTR of *ALAD* with SP	*Homo sapiens*	Donner et al. [111]
miR-212	Pre-miRNA up-regulated expression in the medial pre-frontal cortex following maternal separation	*Rattus norvegicus*	Uchida et al. [87]
miR-221	Decreased expression in the hippocampus due to treatment with lithium and sodium valproate	*Rattus norvegicus*	Zhou et al. [68]
miR-273	Plays a role in neuronal differentiation	*Caenorhabditis elegans*	Chang et al. [121]; Johnston et al. [122]; Johnston et al. [123]
miR-323	Correlation between differential expression of this miRNA and behavioural measures for anxiety in mice	*Mus musculus*	Parsons et al. [91]
miR-339	Associated with panic disorder	*Homo sapiens*	Muiños-Gimeno et al. [107]
miR-376	Up-regulated in CA1 region of hippocampus under acute or chronic stress	*Rattus norvegicus*	Meerson et al. [89]
miR-378	Association between miRNA and behavioural measures (exploration, learning and memory) for anxiety in mice	*Mus musculus*	Parsons et al. [91]
miR-451	Association between miRNA and behavioural measures (exploration, learning and memory) for anxiety in mice	*Mus musculus*	Parsons et al. [91]
miR-485-3p	Significantly associated with hoarding subtype of OCD	*Homo sapiens*	Muiños-Gimeno et al. [52]
miR-488	Associated with panic disorder—repression of *POMC*	*Homo sapiens*	Muiños-Gimeno et al. [107]
miR-491	Associated with panic disorder	*Homo sapiens*	Muiños-Gimeno et al. [107]
miR-509	Association of an allelic variant in the target site for miR-509 in *NTRK3* (ss102661458) with panic disorder—reduction of *NTRK3* repression	*Homo sapiens*	Muiños-Gimeno et al. [52]
miR-765	Association of an allelic variant in the target site for miR-765 in *NTRK3* (*ss102661460*) with panic disorder—reduction of *NTRK3* repression	*Homo sapiens*	Muiños-Gimeno et al. [52]

For a comprehensive list of differentially expressed miRNAs in the hippocampus and central amygdala following acute and chronic stress, refer to Meerson et al. [89]

*NTRK3* neurotrophic tyrosine kinase, receptor, type 3; *RGS2* regulator of G protein signalling 2; *BDNF* brain-derived neurotrophic factor; *HTR2C* 5-hydroxytryptamine (serotonin) receptor 2C; *MAOA* monoamine oxidase A; *GABRA6* gamma-aminobutyric acid A receptor, alpha 6; *CCKBR* cholecystokinin B receptor; *POMC* proopiomelanocortin preproprotein

## Discussion

Regulatory gene regions have recently received attention as major contributors to phenotypic diversity and disease. Investigation of these gene elements in complex disorders, such as anxiety disorders, is critical to understanding the genetic aetiology of these disorders. This review aimed to highlight the importance of miRNAs in anxiety and how such information can be exploited to better understand and treat anxiety disorders. The direct role of miRNAs in anxiety is apparent from literature showing that miRNAs target and regulate stress-related proteins (such as miR-34c that targets CRFR1) and facilitate the stress recovery process [90]. In addition, miRNAs can regulate alternative splicing of stress-related genes (such as miR-183-mediated suppression of SC35 that controls alternative splicing of AChE) and, in doing so, regulate neurotransmission under stressful conditions, providing a link between the molecular and physical stress responses [89]. MiRNAs are also involved in allele-specific regulation of genes that play a role in susceptibility to anxiety disorders (such as *NTK3*) [52].

Numerous studies have shown that miRNA expression is altered in response to stress. Parsons et al. were able to show a clear association between behavioural measures for anxiety and differential expression of specific miRNAs in mice [91]. However, miRNA expression is not only altered in response to prolonged stress but also in response to acute stress. Acute stress results in transient expression change and miRNAs can act as modulators that rapidly translate stressful events to altered protein expression [99]. Furthermore, SNPs in the 3′ UTR of genes that have been associated with anxiety disorders have the potential to alter miRNA target recognition sites and therefore alter the expression of the target gene, ultimately affecting stress responses [108, 109].

Finally, miRNAs not only provide us with information regarding the molecular mechanism underlying the therapeutic effects of certain anxiolytic drugs (miR-34a regulates GRM7 levels after lithium/VPA treatment [68] and miR-16 regulates SERT expression in response to Prozac [116]) but also presents novel therapeutic targets for the treatment of anxiety disorders, either through directly targeting the miRNA itself or by targeting the targets of those miRNAs that have been associated with anxiety disorders.

## Conclusion

MiRNAs are of particular importance in anxiety disorders for a number of reasons. First, miRNAs and their mRNA targets are highly abundant in the CNS, and there is mounting evidence for the role of miRNAs in numerous CNS functions. Second, evidence from various sources, such as animal studies, human studies, in vitro cell culture and computational methods, all point to the involvement of miRNAs in the aetiology of anxiety disorders. Third, there is preliminary evidence for the role of miRNAs as therapeutic targets in anxiety and mood disorders. Lastly, preclinical models illustrate that, by changing the levels of miRNAs, a therapeutically desirable change in anxiety-like behaviours can be achieved [19].

To ensure advances in the field, improvements in analytic tools for miRNA analyses are critical. High-throughput sequencing technologies, together with improved validation tools and prediction algorithms, will aid miRNA target identification and validation. Further research should also focus on how polymorphisms might affect the transcription and structure of pre-miRNAs as well as the expression and processing of mature miRNAs [39]. Functional identification of the biological pathways involved in anxiety and the roles of miRNAs within this complex network is pivotal. Comprehensive understanding of anxiety disorders requires a convergence of data from all sources, including genetics, animal models, clinical assessment, neurophysiology and neuroimaging. Such all-encompassing investigation in conjunction with new therapeutic advances might bring us one step closer to understanding and effectively treating anxiety disorders.
